# Application of *Aspergillus niger* Fumonisin Amine Oxidase (AnFAO) to Detoxify Fumonisin-Contaminated Maize

**DOI:** 10.3390/toxins14080544

**Published:** 2022-08-09

**Authors:** Patrick G. Telmer, Megan J. Kelman, Justin B. Renaud, Mark W. Sumarah, Christopher P. Garnham

**Affiliations:** London Research and Development Centre, Agriculture & Agri-Food Canada, 1391 Sandford Street, London, ON N5V 4T3, Canada

**Keywords:** fumonisins, *Aspergillus*, biotransformation, monoamine oxidase, fungus, mycotoxins, maize, *Pichia pastoris*, DDGS, deamination

## Abstract

Fumonisin mycotoxins are a family of secondary metabolites produced by *Fusarium verticillioides* and related species, as well as some strains of *Aspergillus niger*. Fumonisin contamination of maize is a concern when grown under hot, dry conditions. When present above regulatory levels, there can be effects on animal health. New tools to reduce the toxicity of maize and maize products with high concentrations of fumonisin are needed. Recently, we reported an amine oxidase (AnFAO) from a fumonisin-producing *Aspergillus niger* strain capable of oxidatively deaminating intact fumonisins. In this study, AnFAO was used to reduce intact fumonisin concentrations in milled maize flour, whole kernel maize inoculated with fumonisin-producing *Fusarium verticillioides*, and dried distillers’ grains with solubles (DDGS). The data showed that milled maize flour incubated with 1 µM AnFAO for 1 h resulted in complete deamination of FB_1_ and FB_2_. A greater than 90% reduction in FB_1–3_ concentrations was observed following a simple washing procedure of whole kernel maize in the presence of 1 µM AnFAO for 1 h. Similarly, a ≥86% reduction in FB_1–3_ concentrations was observed in DDGS after 4 h incubation with 1 µM AnFAO. Finally, we engineered the methylotrophic yeast *Pichia pastoris* to produce functional AnFAO in both a secreted and intracellular form. These results support the further development and application of AnFAO as a promising tool to remediate fumonisin-contaminated maize and maize products.

## 1. Introduction

Fumonisins are a class of mycotoxins produced by *Fusarium verticillioides* and related species, mainly on maize [1]. This fungus is ubiquitous in healthy plant tissue; however, when the plant is drought stressed, the disease *Fusarium* kernel rot can result in high concentrations of fumonisins. Insects that infest maize increase plant damage and fumonisin concentrations [2]. While fumonisin consumption is generally low in fully developed market economies, exposures can exceed regulatory levels, particularly in parts of Africa and Latin America [3,4,5,6].

**Figure 1:**
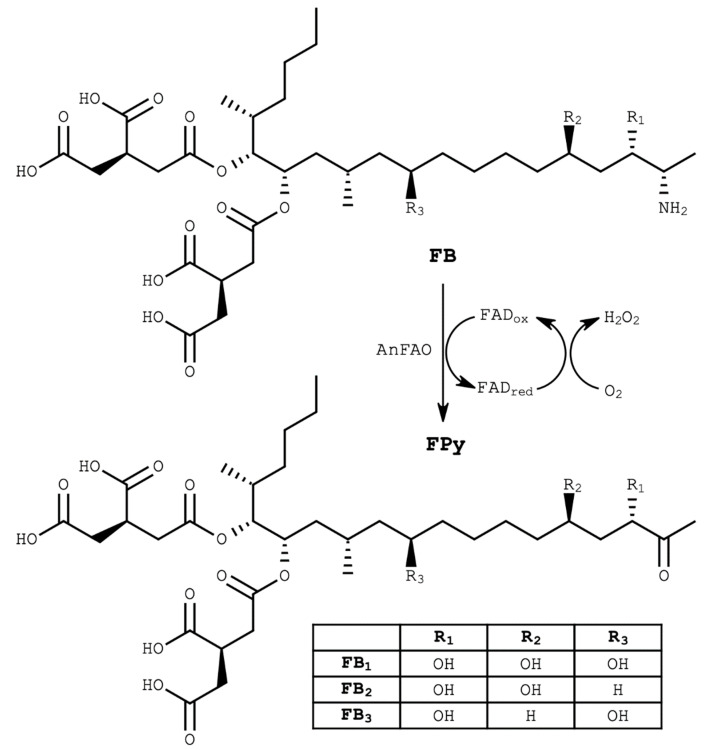
Chemical structure of B-series fumonisins (FB, **top**) and the deaminated FPy form (**bottom**) catalyzed by AnFAO.

Fumonisins are polyketide-derived linear amino polyols that function as competitive inhibitors of the enzyme ceramide synthase [7]. Ingestion of fumonisins results in sphingolipid imbalances that can ultimately lead to programmed cell death [7,8]. Multiple fumonisin congeners exist, with B-series fumonisins (FB) being the most prevalent and toxic. Tricarballylic acid (TCA) and primary amine moieties play predominant roles in the toxicity of the molecule (Figure 1) [8,9].

Some older studies suggest that consumption of fumonisin-contaminated maize is associated with esophageal cancer [10] and neural tube defects in humans [11]. At high exposures, fumonisins can result in leukoencephalomalacia in equine species [12], and at very high exposures, porcine pulmonary edema [13]. There is also some evidence that fumonisin exposures can contribute to child stunting in parts of Africa [5,14]. In 2001, the Joint Expert Committee on Food Additives and Contaminants group established a provisional maximal tolerable daily intake (PMTDI) of 2 µg/kg body weight for FB_1_, FB_2_, and FB_3_, alone or in combination in food, and this PMTDI was reaffirmed in 2018. This is based on liver and kidney toxicity in rats and mice [15]. The US Food and Drug Administration limits for fumonisins in feed for domestic animals range from 5–50 µg/g, depending on the species [16].

Enzymatic biotransformation of mycotoxins can be a rapid, specific, and cost-effective approach to reducing their toxicity. Enzymes that biotransform fumonisins have been previously identified [17,18,19,20]. Enzymatic removal of the TCA side chains via the hydrolase FumD is effective at reducing fumonisin toxicity within animal feed, including whole kernel maize and various end products of maize dry milling [21,22], as well as maize destined for human consumption, and in particular by rural subsistence farming communities [21,22,23]. Enzymes that target the fumonisin amine moiety have also been identified; however, they typically require hydrolyzed fumonisins as substrate and/or also require expensive cosubstrates and cofactors [17,18,19,20]. Additional enzymes that target the fumonisin amine moiety are desirable, as fumonisin deamination is important for reducing the toxicity of the molecule [24].

Recently, we reported an amine oxidase produced by a strain of *Aspergillus niger* capable of deaminating intact fumonisins [25,26]. The enzyme, termed AnFAO (*Aspergillus niger* fumonisin amine oxidase), is an FAD-dependent monoamine oxidase that converts the fumonisin primary amine into an imine, which then spontaneously hydrolyzes to a ketone moiety (Figure 1) [25]. AnFAO does not require exogenous FAD for activity, greatly simplifying the biotransformation process [25]. Toxicity testing with *Lemna minor* demonstrated that oxidized fumonisins containing a ketone in place of the amine are nontoxic to the plant, whereas hydrolyzed fumonisins in the same assay still displayed some residual toxicity [9]. This result is supported by previous work showing that deaminated FB_1_ was much less toxic than intact FB_1_ [24].

In this report, we demonstrate, as a proof of concept, the application of AnFAO as a tool to remediate fumonisin-contaminated maize. In particular, the enzyme was used to reduce the concentrations of intact fumonisins in milled maize flour, whole kernel maize, and dried distillers’ grains with solubles (DDGS), the protein-rich end-feed product of bioethanol production in which fumonisins become significantly enriched when present in the source maize [27,28,29]. We also engineered the methylotrophic yeast *P. pastoris* to produce both intracellular and secreted forms of the enzyme to demonstrate the industrial applicability of the enzyme.

## 2. Results

### 2.1. Recombinant AnFAO Deaminates Intact Fumonisins in QCM Milled Maize Flour

**Figure 2:**
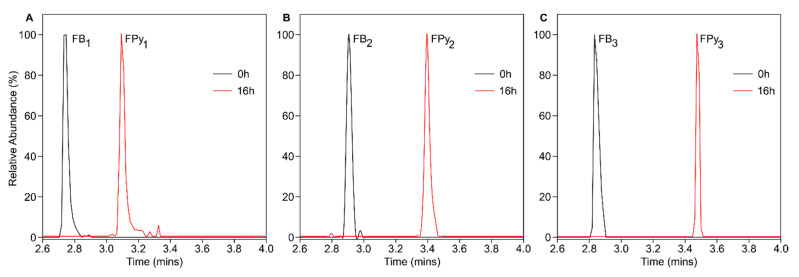
LC-MS extracted ion chromatograms ([M − H]^−^; ±3 ppm) of QCM milled maize flour treated with 1 µM AnFAO at room temperature overnight. Complete conversion of intact FB to deaminated (FPy) forms was observed for FB_1_ (**A**), FB_2_ (**B**), and FB_3_ (**C**). Black line traces represent t = 0 h, while red line traces represent t = 16 h.

QCM milled maize flour containing a mixture of fumonisins was reconstituted in Milli-q H_2_O. AnFAO was added to a final concentration of 1 µM and incubated overnight at room temperature. LC-MS analysis of the reaction indicated that intact fumonisins were converted into their deaminated counterparts (Figure 2).

**Figure 3:**
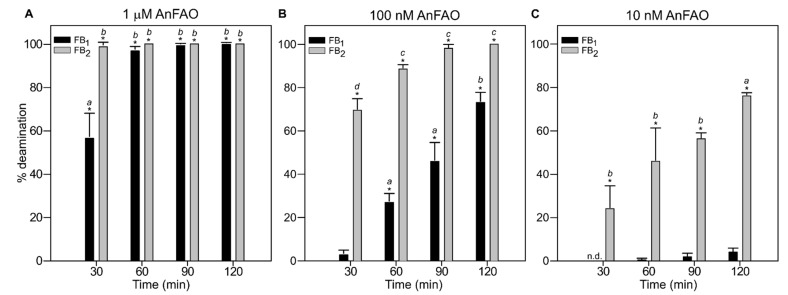
Percent fumonisin deamination as a function of time and [AnFAO] within QCM milled maize flour as determined via LC-MS analysis: (**A**) 1 µM AnFAO; (**B**) 100 nM AnFAO; and (**C**) 10 nM AnFAO. Error bars represent standard error (*n* = 3). * indicates that the mean is significantly different (*p* < 0.01) from the 0 min time point. Data marked with unique italicized letters are significantly different from each other at *p* < 0.01 (Tukey’s multiple comparison test). n.d. indicates not determined due to LC-MS signal level being below the detection limit of the instrument.

These results demonstrate that purified recombinant AnFAO was capable of deaminating fumonisins within a complex aqueous feed matrix and that no additional reagents or cofactors were required. To determine the optimal enzyme concentration and incubation time required for fumonisin conversion within this mixture, a time course was conducted at varying AnFAO concentrations (Figure 3). At 1 µM AnFAO, FB_1_ was fully deaminated after 60 min, while FB_2_ was fully deaminated after 30 min (Figure 3A). No further significant increases in deamination were observed beyond 30 and 60 min for FB_1_ or FB_2_, respectively. In addition, we observed significantly greater deamination of FB_2_ compared with FB_1_ at the 30 min time point, with only ca. 56% of FB_1_ being deaminated compared with complete deamination of FB_2_. (Figure 3A). At 100 nM AnFAO, we observed ca. 69%, 88%, and 98% deamination of FB_2_ following 30, 60, and 90 min treatments, respectively, with no further significant increase in deamination at the 2 h time point (Figure 3B). For FB_1_, we observed ca. 3%, 27%, 46%, and 73% deamination following 30, 60, 90, and 120 min treatments, respectively. The percent deamination of FB_2_ compared with FB_1_ using 100 nM AnFAO was significantly greater at every time point (Figure 3B), with the greatest difference coming after 30 min (ca. 69% FB_2_ vs. 3% FB_1_). At 10 nM AnFAO, we observed 24%, 46%, 56%, and 76% deamination of intact FB_2_ following 30, 60, 90, and 120 min treatments, respectively. We observed only minimal deamination of FB_1_, with ca. 4% of intact FB_1_ being converted to FPy_1_ after 2 h. (Figure 3C). We again observed significantly greater deamination of FB_2_ at every time point compared with FB_1_ when using 10 nM AnFAO.

### 2.2. AnFAO Deaminates Intact Fumonisins in F. verticillioides-Contaminated Whole Kernel Maize

**Figure 4:**
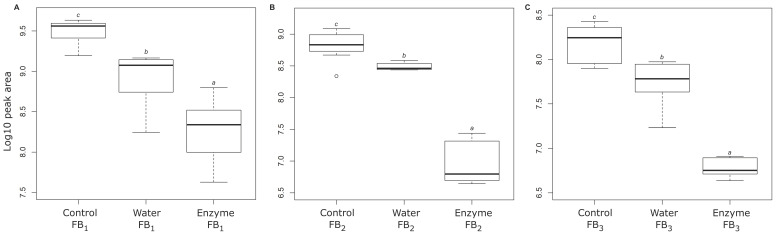
Box and whisker plot of LC-MS peak area (log_10_) of (**A**) FB_1_, (**B**) FB_2_, and (**C**) FB_3_ in *F. verticillioides*-contaminated maize kernels following control, water, and AnFAO treatment (*n* = 3 for all conditions). Compounds bearing unique italicized letters are significantly different by Tukey’s test (*p* < 0.05).

The ability of AnFAO to deaminate fumonisins was examined in whole kernel maize infected with *F. verticillioides*. Culture material was mixed with clean kernels at a 1:10 ratio in order to simulate naturally occurring “hot” spots [30]. LC-MS analysis of maize kernel extracts prior to treatment with AnFAO indicated an average of 34 ppm FB_1_, 8 ppm FB_2_, and 4 ppm FB_3_. Prior to the addition of AnFAO, the maize was washed with water at room temperature for 60 min following published protocols (Figure 4) [30]. As expected, washing with water significantly lowered the levels of all fumonisins present in the maize, and in particular, FB_1_ levels decreased by 69%, FB_2_ levels decreased by 60%, and FB_3_ levels decreased by 64%. The addition of 1 µM AnFAO to the wash mixture further significantly reduced the levels of intact FB_1_, FB_2_, and FB_3_ by 92%, 98%, and 96%, respectively (Figure 4).

### 2.3. AnFAO Deaminates Fumonisins within DDGS

**Figure 5:**
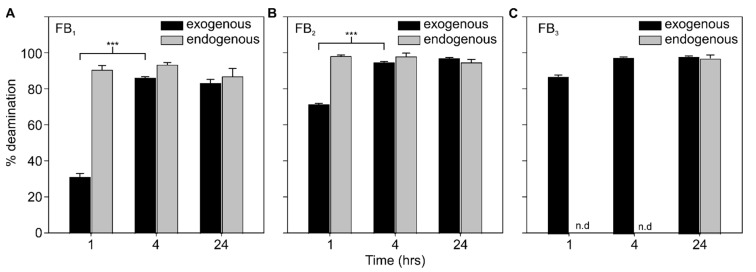
AnFAO deaminates fumonisins within DDGS. LC-MS analysis monitoring % deamination of (**A**) FB_1_, (**B**) FB_2_, and (**C**) FB_3_ exogenously spiked into DDGS (black bars) or naturally present in the sample (gray bars). Error bars represent standard error (*n* ≥ 3). n.d. indicates not determined due to LC-MS signal level being below the detection limit of the instrument. *** indicates a statistically significant difference between means at 1 and 4 h time points. (*p* < 0.001).

DDGS was incubated with 1 µM AnFAO and monitored for fumonisin deamination over time by LC-MS. Native fumonisin levels of FB_1_, FB_2_, and FB_3_, as determined via LC-MS, were at 14, 2, and 1 ppb, respectively. FB_1_, FB_2_, and FB_3_ were added to reach concentrations of 2, 0.47, and 0.27 ppm, respectively, ca. 3× the concentrations present within the QCM milled maize flour used in this study. These values were selected to replicate the enrichment in DDGS that typically occurs during bioethanol production [27]. Significant fumonisin deamination was observed during incubation with AnFAO (Figure 5). In particular, approximately 31% of FB_1_ was deaminated after 1 h, while 71% of FB_2_ and 87% of FB_3_ were deaminated in the same time frame. Further incubation up to 4 h significantly increased deamination to 86% for FB_1_ and 95% for FB_2_, while FB_3_ deamination increased to 97%. However, this increase was not statistically significant compared with the 1 h time point. Additional incubation up to 24 h did not result in further significant increases in activity, with % deamination remaining at 83% for FB_1_, 97% for FB_2_, and 98% for FB_3_. In the absence of added fumonisins, AnFAO effectively deaminated the native fumonisins present within the DDGS sample. Greater than 90% deamination of all fumonisins after 1 h incubation was observed, with no additional removal at the later time points. The remaining FB_3_ could not be quantified due to low natural abundance.

### 2.4. Pichia Pastoris Produces Active AnFAO in Both Secreted and Intracellular Forms

**Figure 6:**
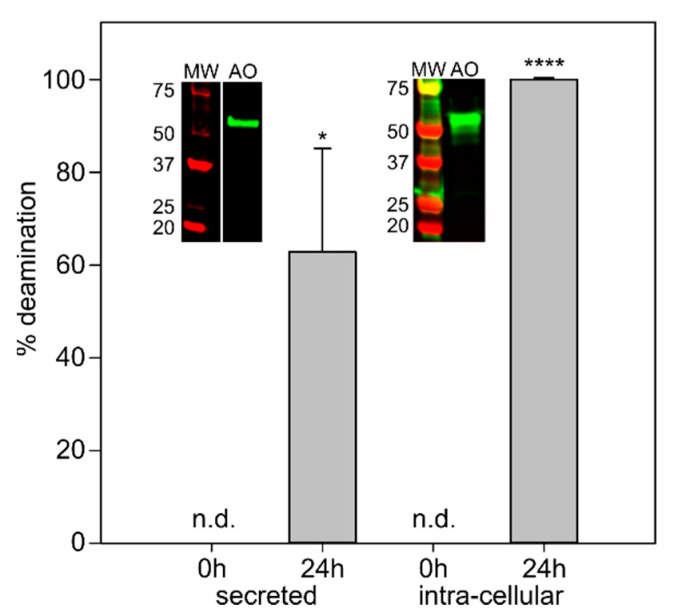
*Pichia pastoris* produces active AnFAO. Percent fumonisin deamination of FB_2_ as monitored via LC-MS by secreted and intracellularly retained forms of AnFAO. The *x*-axis represents hours of recombinant protein production post methanol induction. Error bars represent standard deviation (*n* = 3). n.d. indicates no activity detected. Insets represent Western blots of either the *P. pastoris* culture supernatant (left) or crude cell lysate (right) probed with an anti6x-His antibody. MW indicates protein molecular weight markers, and AO indicates amine oxidase. * and **** indicate the mean is significantly different from the 0 h time point (*p* < 0.05 and <0.0001, respectively).

The methylotrophic yeast *P. pastoris* produced AnFAO both intracellularly and in a secreted form. Following 24 h of methanol-induced protein expression, we tested both the culture supernatant of the secreted clone and the clarified cell lysate of the intracellular clone for fumonisin deamination activity. Both samples were incubated with 1 µM FB_2_ for 16 h at 30 °C and analyzed via LC-MS for fumonisin deamination activity (Figure 6). The secreted and intracellular clones of AnFAO deaminated 65% and 100% of the 1 µM intact FB_2_, respectively. Neither *Pichia* strain bearing the secreted or intracellular constructs were capable of deaminating fumonisins prior to methanol-induced protein expression. Western blotting using an anti6x-His-tag antibody showed a clear signal at the correct molecular weight (secreted = 63.7 kDa; intracellular = 54.1 kDa) for both the secreted and intracellular forms of AnFAO. These results indicate that the yeast *P. pastoris* can produce recombinant AnFAO that is active and capable of deaminating fumonisins in a complex matrix without the need for exogenous FAD cofactor.

## 3. Discussion

The present studies demonstrate the practicality and broad applicability of AnFAO as a potential biotransformation tool for fumonisins contaminated food and feed. AnFAO significantly deaminated fumonisins in QCM milled maize flour contaminated with known amounts of the toxins (Figure 2 and Figure 3) and removed significantly more fumonisins from maize contaminated with a fumonisin-producing strain of *F. verticillioides* than washing protocols alone (Figure 4). AnFAO also effectively decreased the concentration of intact fumonisins in DDGS (Figure 5) and was active in both intracellular and secreted forms following expression in the methylotrophic yeast *P. pastoris* (Figure 6).

While the risk from fumonisins to human populations is low in most developed countries, populations that rely on a high percentage of maize in the diet face health risks from consuming contaminated grains [23,30,31]. Previous studies have shown that simply sorting and washing maize reduced fumonisin contamination by 84%, with sorting contributing to a 71% reduction and washing further reducing fumonisin levels by an additional 13% [30]. This reduction led to an estimated probable daily intake (PDI) of fumonisins of 1.4 µg/kg body weight/day, below the JECFA recommended limit of 2 µg/kg body weight/day [32]. Our results demonstrate that relatively high levels of fumonisin contamination (34 ppm FB_1_, 8 ppm FB_2_, and 4 ppm FB_3_) can be reduced by a simple washing step, as has been previously reported [30,31], and can be further enhanced when washing is supplemented with AnFAO. Washing alone reduced FB_1_ levels by 69%, while supplementation with AnFAO brought total reduction to 92%. Similarly, FB_2_ and FB_3_ levels dropped 60% and 64%, respectively, by washing alone, while the addition of AnFAO brought the total reduction to 98% and 92%, respectively (Figure 4).

Our whole kernel maize deamination experiments were performed using 1 µM AnFAO, which represents ca. 10 units of enzyme activity per liter of wash solution. These results are similar to those obtained by Alberts et al. (2019), who observed significant decreases in intact FB_1_ levels and increases in hydrolyzed FB_1_ levels beginning at 10 U/L of the fumonisin esterase FumD when supplemented in FB-contaminated homegrown maize [21].

AnFAO was also effective at deaminating fumonisins within DDGS. FB_1_, FB_2_, and FB_3_ were added at concentrations commonly encountered in DDGS samples isolated from various bioethanol production plants [27]. We observed robust (>80%) deamination of all intact fumonisin congeners after 4 h incubation with 1 µM AnFAO (Figure 5). The naturally occurring fumonisin concentration in our particular DDGS sample was low but still quantifiable. AnFAO was also effective at deaminating these fumonisins present within the sample, with all FBs undergoing >90% deamination after 1 hour, demonstrating that enzymatic access to fumonisins within DDGS is not limiting.

The production of AnFAO in a yeast platform is critical for demonstrating the commercial potential of this enzyme with respect to animal feed and applications in bioethanol production. The secreted form of AnFAO produced by *P. pastoris* deaminated FB_2_ administered directly to the culture supernatant without the need for affinity tag enrichment or additional FAD cofactor. The intracellular form was also active and capable of deaminating FB_2_ within the crude cellular lysate (Figure 6). Production of AnFAO within a yeast platform will enable the evaluation of AnFAO as a tool to treat fumonisin-contaminated DDGS [33].

Several enzymes have been documented that biotransform fumonisins into less toxic forms. *Sphingopyxis* sp. MTA144 produces the carboxylesterase (FumD) and aminotransferase (FumI) that remove fumonisin TCA side chains and amine moiety, respectively [18]. FumI requires the hydrolyzed fumonisin end product of FumD as a substrate and also requires pyruvate and pyridoxal phosphate as a cosubstrate and cofactor, respectively [17]. The black yeast *Exophiala spinifera* produces an FAD-dependent amine oxidase similar to AnFAO [19,20]. Similar to FumI, the wild-type form of the enzyme also requires the removal of the TCA side chains with an upstream enzyme prior to oxidative deamination. Our recent toxicology data using duckweed as the host indicates that the removal of the amine moiety from fumonisins is a key mechanism for reducing the toxicity of the molecule [9]. Our results demonstrate that AnFAO can be produced in multiple recombinant systems, including *E. coli* and *P. pastoris,* at significant yields and does not require exogenous cofactors or additional upstream enzymes for activity. These characteristics make the enzyme an ideal tool for fumonisin deamination in real-world settings, including potentially during bioethanol production, pelletization in animal feed, and direct addition to wash solutions. Furthermore, AnFAO is not predicted to be allergenic [25] and is derived from *Aspergillus niger*. Many strains of this fungus have been granted US GRAS status (generally regarded as safe) and are widely used as an industrial production platform for important enzymes and chemicals [34].

AnFAO demonstrates modest affinity and catalytic efficiency for fumonisins (K_M_ = 390.6 µM and k_cat_ = 8.7 min^−1^ for FB_1_, K_M_ = 194.7 µM and k_cat_ = 13 min^−1^ for FB_2_) [25]. Despite these relatively modest numbers, our data indicate the enzyme is highly effective at reducing fumonisin contamination in multiple food and feed sources. Future studies will focus on understanding the structure:function relationship of the enzyme in order to identify key residues involved in mediating specificity with the substrate and, therefore, identifying and designing new variants of the enzyme with improved catalytic activity.

In conclusion, the data provided demonstrated that AnFAO is capable of reducing the concentrations of intact fumonisins in milled maize flour, whole kernel maize infected with *F. verticillioides*, and DDGS. Deamination with AnFAO is a simple process that does not require additional cofactors or upstream enzymes. AnFAO could be an effective tool for fumonisin deamination in animal feed in developed countries and in food in developing countries that are at high risk of fumonisins exposure.

## 4. Materials and Methods

### 4.1. Chemicals, Expression Vectors, and Reagents

Multitoxin quality control material (QCM) in milled maize flour was obtained from Romer Labs (Newark, DE, USA) (Cat. No. QCM7C1) (initial fumonisin levels µg/kg: 667 ± 78 FB_1_, 156 ± 21 FB_2_, and 89 ± 22 FB_3_). FB_1_ and FB_2_ were obtained from Sigma-Aldrich (St. Louis, MO, USA) (Cat. No. F7817 and SML128: 1 mg/mL in DMSO). FB_3_ was obtained from Romer Labs as a 50.7 µg/mL solution in acetonitrile (SH-FUM B3). Protein reagents including β-mercaptoethanol, isopropyl β-D-1-thiogalactopyranoside (IPTG), and SDS-PAGE gels and supplies were obtained from BIO-RAD (Mississauga, ON, Canada). Luria Bertani (LB) growth media, buffers, salts, and all other general reagents were obtained from either Sigma-Aldrich, Fisher Scientific (Ottawa, ON, Canada), or VWR (Mississauga, ON, Canada).

### 4.2. LC-MS Analysis of Fumonisins

LC-MS data were acquired with a Q-Exactive^TM^ Quadrupole Orbitrap mass spectrometer (Thermo Scientific, Waltham, MA, USA) coupled to an Agilent 1290 ultrahigh-performance liquid chromatography (UHPLC) system as previously described [25]. A total of 5 µL of each extracted sample was injected onto a Zorbax Eclipse Plus RRHD C18 (2.1 mm × 50 mm, 1.8 µm; Agilent Technologies, Santa Clara, CA, USA) column and maintained at 35 °C. The mobile phase comprised 0.1% formic acid (A) and acetonitrile with 0.1% formic acid (B) (Optima grade, Fisher Scientific, Somerville, NJ, USA). The gradient consisted of 0% B for 0.5 min before increasing to 100% over 3 min, held at 100% for 2.5 min, and reduced to 0% over 0.5 min. Fumonisins were detected in negative ionization mode using the following electrospray conditions: capillary voltage, 4.0 kV; capillary temperature, 400 °C; sheath gas, 17.00 units; auxiliary gas, 8.00 units; probe heater temperature, 450 °C; S-Lens RF level, 45.00. The data-dependent acquisition method involved a full MS scan at 17,500 resolutions over a scan range of 140–760 *m*/*z*; the automatic gain control (AGC) target and maximum injection time (max IT) were 5 × 10^6^ and 64 ms, respectively. The five highest intensity ions from the full scan (excluding isotopes) were sequentially selected using a 1.2 *m/z* isolation window and analyzed at a resolution of 17,500; AGC target, 1 × 10^5^; max IT, 64 ms; normalized collision energy (NCE), 30; threshold intensity, 9.1 × 10^4^; and dynamic exclusion, 1.5 s. Fumonisins were detected by accurate mass (±5 ppm) and retention time (±0.1 min) and verified by MS/MS.

### 4.3. AnFAO Expression and Purification

Recombinant AnFAO was expressed in *E. coli* BL21DE3 as an N-terminal 6X His-tagged MBP-fusion protein (Addgene plasmid #29656, a gift from Scott Gradia) and purified to homogeneity via metal affinity, hydrophobic interaction, and gel permeation chromatography steps using a Bio-Rad FPLC system as previously described [25]. This purified recombinant form of the enzyme was employed in the QCM milled maize flour, whole kernel maize, and DDGS experiments.

### 4.4. Deamination of Fumonisins in QCM Milled Maize Flour

In total, 150 mg of QCM containing known levels of fumonisins in milled maize flour was mixed with 500 µL Milli-q H_2_O. Purified recombinant AnFAO was added to the mixture at concentrations of 10 nM, 100 nM, and 1 µM. Samples were mixed by vortexing for 30 s and incubated at RT with shaking for intervals from 30 min up to 16 h total. At specified time points, fumonisins were extracted from each sample as follows: 700 µL of 78% acetonitrile, 2% acetic acid, and 20% Milli-q H_2_O was added to each sample and incubated at 37 °C with vigorous shaking for 1 h. Samples were then subject to centrifugation at 20,000× *g*, and 500 µL of this supernatant was removed and mixed with an equal volume of 100% methanol. The solution was then mixed by inversion and centrifuged at 20,000× *g* to remove insoluble debris prior to LC-MS analysis. Five µL of each sample was analyzed by LC-MS. Percent deamination was calculated by measuring calibrated peak areas for each intact and deaminated fumonisin and dividing the amount of deaminated fumonisin by the total amount of fumonisin [25,26]. Tukey’s multiple comparison test was performed to detect differences between means using the statistical tools in Sigmaplot (*p* < 0.01).

### 4.5. Deamination of Fumonisins on Whole Kernel Maize Infected with F. verticillioides

Feed maize was obtained from a local mill, and 1 kg was imbibed overnight in a 2 L Erlenmeyer flask with 500 mL Milli-q H_2_O. Excess water was decanted from the kernels prior to autoclave sterilization.

An agar plug (approximately 7 cm × 4 cm) from a 7-day-old culture of *Fusarium verticillioides* (DAOM 250206) grown on Potato Dextrose Agar (Sigma-Aldrich) was aseptically macerated with a polytron blender in 200 mL sterile distilled water. The fungal inoculant was slowly poured over the sterilized maize kernels while swirling the flask to ensure the kernels were coated. The inoculated maize kernels were then grown in darkness at 28 °C for 2 weeks. Infected kernels were oven dried at 40 °C for 4 h and were stored in the freezer (−20 °C) until use. Then, 900 g of clean feed maize was fortified with ca. 100 g of the highly contaminated material to produce the experimental maize used to perform subsequent enzyme fumonisin conversion analyses [30]. The experimental maize was quantified by LC-MS to have 34, 8, and 4 µg/g of FB_1_, FB_2_, and FB_3_, respectively.

Approximately 10 g of *F. verticillioides*-contaminated whole maize kernels were placed in sterile 50 mL Erlenmeyer flasks in triplicate, whereupon 10 mL of Milli-q H_2_O was added to each. Purified recombinant AnFAO was then added to a final concentration of 1 µM and incubated at RT with gentle shaking for 60 min. The water and enzyme-washed kernels were then air-dried at RT for 30 min prior to milling and fumonisin extraction and LC-MS analysis (as described previously for QCM flour extraction). Extracted peak areas of FB_1_, FB_2_, and FB_3_ for the water and enzyme washes were compared with the experimental control maize samples. Single-factor ANOVA was performed using Tukey’s post hoc test (*p* < 0.05) using the log 10 peak areas between treatment groups using the *dplyr* and *multcomp* statistical analysis packages in R. All box and whisker plots were produced using R.

### 4.6. Deamination of Fumonisins in Dried Distillers’ Grains with Solubles (DDGS)

DDGS were obtained from a local bioethanol production facility and were stored at −20 °C prior to use in the experiment. FB_1_, FB_2_, and FB_3_ were dissolved in 100% methanol and added to 100 mg of DDGS to a final concentration of 2.0, 0.47, and 0.27 ppm of FB_1_, FB_2_, and FB_3_, respectively [27]. This mixture was allowed to dry prior to mixing with 500 µL ddH_2_O. Purified recombinant AnFAO was added to the mixture at a concentration of 1 µM. Control reactions included: (1) samples containing known concentrations of “spiked” fumonisins without enzyme and (2) samples of DDGS containing only native levels of fumonisins (non-spiked samples) incubated with AnFAO. Samples were mixed by vortexing for 30 s and incubated at RT with shaking for intervals of 1, 4, and 24 h. At the specified time points, fumonisins were extracted from each sample as described for the QCM experiments. Percent deamination was calculated as described above. Statistically significant differences between the 1 and 4 h time points for exogenous fumonisins were detected by *t*-test (*p* < 0.0001) using Sigmaplot.

### 4.7. Recombinant Production of AnFAO in the Methylotrophic Yeast Pichia pastoris

AnFAO was synthesized and codon-optimized for expression in *Pichia pastoris* by Gene Universal Ltd. The gene was subsequently cloned into the *Eco*RI and *Not*I restriction sites of pPICZαA and pPICZB vectors to enable secreted and intracellular expression, respectively, within the methylotrophic yeast *Pichia pastoris,* as per the manufacturer’s instructions (Life Technologies *Pichia* Expression kit). Approximately 10 µg of either pPICZαA-AnFAO or pPICZB-AnFAO were transformed into *Pichia pastoris* strain X33 via electroporation. Transformants were plated onto YPDS (1% yeast extract, 2% peptone, 2% dextrose, 18.2% sorbitol) agar plates containing 100 µg/mL Zeocin and incubated for 48 h at 30 °C. Colonies were picked and spotted onto YPDS plates containing 1 mg/mL of Zeocin and incubated for 48 h at 30 °C. Colonies from each construct were then used to inoculate 10 mL of BMGY liquid media (2.0% peptone, 1.0% yeast extract, 100 mM potassium phosphate (pH 6.0), 1.34% yeast nitrogen base (without amino acids), 0.4 µg/mL biotin, 1.0% glycerol) containing 100 µg/mL Zeocin and placed in a shaking incubator for 16 h at 30 °C. Cells were harvested by centrifugation and washed 2× with 10 mL of BMMY liquid media (same as BMGY media except containing 0.5% methanol as carbon source instead of 1.0% glycerol). The cells were then suspended at an OD_600_ of 0.8–1 in 50 mL of BMMY (without Zeocin), transferred to sterile baffled flasks, and grown continuously at 30 °C with vigorous shaking (250 rpm). Samples were taken at 24 h post-methanol induction and were assayed for fumonisin deamination activity via LC-MS as follows: Cultures of the secreted version of AnFAO (pPICZαA-AnFAO) were subject to centrifugation, and the cell-free conditioned media was incubated with 1 µM FB_2_ for 16 h at 30 °C before extraction with 50% methanol and analysis by LC-MS. For the non-secreted construct (pPICZB-AnFAO), the cell pellet was lysed in 1 mL phosphate-buffered saline (PBS) (pH 7.4) containing 0.1 mM PMSF and 200 units/mL lyticase using an equal volume of acid-washed glass beads and six rounds of vortexing and incubation on ice. The cell lysate was cleared by centrifugation at 21,000× *g*, and the supernatant was incubated with 1 µM FB_2_ at 30 °C for 16 h before methanol extraction and LC-MS analysis. To check for protein expression, the culture supernatant from the secreted construct (pPICZαA-AnFAO) was concentrated 10-fold using a 1 mL Amicon membrane concentrator (30 kDa cutoff) and subjected to SDS-PAGE followed by immunoblotting using an anti6x-His antibody to detect the N-terminal 6x histidine tag. The cell lysate from the non-secreted construct was subjected to SDS-PAGE and Western blotting in the same manner as described for pPICZαA-AnFAO to detect the recombinant protein. Statistically significant differences between means at the 0 and 24 h time points were detected by *t*-test using Sigmaplot.

## Data Availability

All data available upon request.
